# Heightened sympathetic neuron activity and altered cardiomyocyte properties in spontaneously hypertensive rats during the postnatal period

**DOI:** 10.3389/fnsyn.2022.995474

**Published:** 2022-09-30

**Authors:** Marián Haburčák, Joshua Harrison, Melda M. Buyukozturk, Surbhi Sona, Samuel Bates, Susan J. Birren

**Affiliations:** ^1^Biology Department, Brandeis University, Waltham, MA, United States; ^2^Volen Center for Complex Systems, Brandeis University, Waltham, MA, United States; ^3^Genomic Medicine, Lerner Research Institute, Cleveland Clinic, Cleveland, OH, United States; ^4^Department of Nutrition, Center for Proteomics and Bioinformatics, School of Medicine, Case Western Reserve University, Cleveland, OH, United States

**Keywords:** sympathetic neuron, cardiomyocyte, postnatal, co-culture, SCG, SHR, excitability, synaptic transmission

## Abstract

The Spontaneously Hypertensive Rat (SHR) has increased sympathetic drive to the periphery that precedes and contributes to the development of high blood pressure, making it a useful model for the study of neurogenic hypertension. Comparisons to the normotensive Wistar Kyoto (WKY) rat have demonstrated altered active and intrinsic properties of SHR sympathetic neurons shortly before the onset of hypertension. Here we examine the structural and functional plasticity of postnatal SHR and WKY sympathetic neurons cultured alone or co-cultured with cardiomyocytes under conditions of limited extrinsic signaling. SHR neurons have an increased number of structural synaptic sites compared to age-matched WKY neurons, measured by the co-localization of presynaptic vesicular acetylcholine transporter and postsynaptic shank proteins. Whole cell recordings show that SHR neurons have a higher synaptic charge than WKY neurons, demonstrating that the increase in synaptic sites is associated with increased synaptic transmission. Differences in synaptic properties are not associated with altered firing rates between postnatal WKY and SHR neurons and are not influenced by interactions with target cardiomyocytes from either strain. Both SHR and WKY neurons show tonic firing patterns in our cultures, which are depleted of non-neuronal ganglionic cells and provide limited neurotrophic signaling. This suggests that the normal mature, phasic firing of sympathetic neurons requires extrinsic signaling, with potentially differential responses in the prehypertensive SHR, which have been reported to maintain tonic firing at later developmental stages. While cardiomyocytes do not drive neuronal differences in our cultures, SHR cardiomyocytes display decreased hypertrophy compared to WKY cells and altered responses to co-cultured sympathetic neurons. These experiments suggest that altered signaling in SHR neurons and cardiomyocytes contributes to changes in the cardiac-sympathetic circuit in prehypertensive rats as early as the postnatal period.

## Introduction

The sympathetic nervous system plays a critical role in the regulation of blood pressure, with enhanced sympathetic drive observed before the onset of hypertension in both human and animal models of the disorder (Grassi et al., 2015; Davis et al., 2020). The sympathetic circuit is composed of central and peripheral components, with the peripheral sympathetic ganglia providing the interface between these systems. Postganglionic sympathetic neurons within discrete ganglia along the sympathetic chain receive central inputs from cholinergic preganglionic spinal cord neurons residing in the intermediolateral column and, in turn, project to peripheral target structures that include the heart, kidneys, and vasculature (Coote and Chauhan, 2016).

The level of peripheral sympathetic activity is set both by the strength of the central inputs driving ganglionic sympathetic neuron activity (McLachlan et al., 1998; Bratton et al., 2010), and by the output properties of the ganglionic neurons themselves (Springer et al., 2015; McKinnon et al., 2019). Sympathetic neuron activity is regulated by local ganglionic and peripheral factors, including target-derived neurotrophic factors that modulate the intrinsic and synaptic properties of sympathetic output neurons (Luther et al., 2013; Lehigh et al., 2017; Enes et al., 2020). These regulatory factors contribute to the development and regulation of normal sympathetic function, and may, when dysregulated, contribute to sympathetic pathologies such as hypertension.

Spontaneously Hypertensive Rats (SHR) provide a well-established model for studying neurogenic aspects of essential hypertension (Okamoto and Aoki, 1963; Doris, 2017). The multigenic, multifactorial hypertension that develops in the SHR recapitulates common characteristics of hypertension in the human population (Grassi et al., 2015; Doris, 2017), including persistent alterations in sympathetic function (Dornas and Silva, 2011). Heightened sympathetic activity in adult SHRs has been documented as increases in ganglionic output (Magee and Schofield, 1992) and catecholamine release (Ekas and Lokhandwala, 1981; Westfall and Meldrum, 1985) and altered sympathetic properties are seen at multiple sites of sympathetic innervation including in the heart (Shanks et al., 2013), kidney (De Michele and Amenta, 1988; Kapuscinski et al., 1996) and the cerebral vasculature (Mangiarua and Lee, 1990) when compared to Wistar Kyoto (WKY) rats. The finding that sympathectomy reduces blood pressure in adult hypertensive SHRs provides further evidence of the contributions of the sympathetic system to hypertension in these rats (Brock et al., 1996).

Changes in sympathetic properties are seen in the prehypertensive period for both humans at risk for hypertension (Lopes et al., 2000) and in the SHR. SHR sympathetic neurons show increased excitability 2–3 weeks before the onset of high blood pressure (Davis et al., 2020). SHR neurons also show altered calcium homeostasis (Li et al., 2012) and an increase in cardiac sympathetic drive (Shanks et al., 2013) in the prehypertensive period. Prehypertensive changes have been observed in both the stellate ganglia (Shanks et al., 2013), which provide the large majority of cardiac innervation to the heart (Pardini et al., 1989) and the superior cervical ganglia (SCG; Shanks et al., 2013; Martinez et al., 2019), which project to targets in the head and neck, with a minor projection to the heart (Pardini et al., 1989). Further, sympathetic lesions in neonatal SHRs block the later development of high blood pressure, suggesting that early changes in sympathetic properties may contribute to the later onset of hypertension (Lee et al., 1987).

Sympathetic ganglion neurons in the rat develop and function in a complex environment (Scott-Solomon et al., 2021) that includes cholinergic spinal cord preganglionic terminals (McLachlan et al., 1998), close contact with sympathetic satellite glia within the ganglia (Enes et al., 2020; Mapps et al., 2022), and feedback signaling from cardiomyocytes (Lockhart et al., 2000; Moon and Birren, 2008; Dokshokova et al., 2022). The properties of SHR sympathetic neurons have generally been studied in the context of that complex milieu, often in *ex vivo* or cell culture preparations containing high neurotrophin levels and multiple cell types (Yarowsky and Weinreich, 1985; Magee and Schofield, 1994; Shanks et al., 2013). Here we examine the properties of postnatal SHR sympathetic neurons in a culture system in which we deplete non-neuronal cells and use a minimally sufficient NGF level for neuronal survival (Lockhart et al., 1997) to allow us to examine neuronal properties in a system that minimizes extrinsic signaling. In these cultures, sympathetic neurons develop a spontaneously active cholinergic network that can be used as a model for cholinergic inputs from the spinal cord (O’Lague et al., 1974; Gingras et al., 2002; Vega et al., 2010), even as they co-express noradrenergic properties (Lockhart et al., 1997; Slonimsky et al., 2003). We show increased synaptic properties of SHR neurons in comparison to WKY neurons that are not further enhanced by co-culture with cardiomyocytes. In contrast to neurons cultured under more complex conditions (Jubelin and Kannan, 1990; Davis et al., 2020), we observe tonic firing of both WKY and SHR neurons. Further, we find size differences between postnatal SHR and WKY cardiomyocytes and altered responses to co-cultured neurons. These findings suggest that SHR sympathetic neurons have increased synaptic capacity as early as the postnatal period and that both neurons and cardiomyocytes in the SHR sympathetic-cardiac circuit have altered responses to cellular signals that developmentally set excitability and cardiomyocyte properties.

## Methods

### Cell cultures

All experimental procedures involving animals were approved by the Brandeis University Institutional Animal Care and Use Committee. Superior cervical sympathetic ganglia (SCG) and hearts were dissected from P2-P4 SHR and WKY rats and were used to generate primary cultures of sympathetic neurons and/or cardiac myocytes.

*Primary neuronal cultures* were generated by incubating isolated de-sheathed ganglia for 1 h at 37°C in minimum essential medium for suspension cultures (S-MEM, Gibco BRL, Invitrogen, Carlsbad, CA, USA) containing 350 units/ml collagenase type I (Worthington Biochemical Corporation, Lakewood, NJ, USA) and 7.5 units/ml dispase (Gibco BRL, Invitrogen, Carlsbad, CA, USA). Cells were dissociated by multiple passes through a fire-polished glass pipette, and adherent non-neuronal cells were removed by pre-plating dissociated cells on uncoated plastic tissue culture dishes for 1 h at 37°C. The less adherent aggregates of neurons and satellite glia, were rinsed off the dishes and plated at a density of 10,000 cells per dish on glass-bottomed 35 mm dishes (MatTek Corporation, Ashland, MA, USA) coated with collagen (50 μg/ml; BD Biosciences, Bedford, MA, USA) and mouse laminin (5 μg/ml; BD Biosciences, Bedford, MA). Cultures were maintained in a modified L15CO_2_ medium (Hawrot and Patterson, 1979; Lockhart et al., 1997), supplemented with 10% fetal bovine serum (Gibco BRL, Invitrogen, Carlsbad, CA, USA), 6 μg/ml dextrose, 2 mM glutamine (Invitrogen, Carlsbad, CA, USA), 100 units/ml penicillin and 100 μg/ml streptomycin (Invitrogen, Carlsbad, CA, USA), 1 μg/ml 6,7, dimethyl-5, 6,7,8-tetrahydropterine (DMPH4, Calbiochem, San Diego, CA, USA), 5 μg/ml glutathione (Sigma, St. Louis, MO, USA) and 100 μg/ml L-ascorbic acid. Mouse 2.5S NGF (nerve growth factor, 5 ng/ml, Alomone Labs, Jerusalem, Israel) was added to all cultures to support neuronal survival. Cytosine arabinofuranoside (AraC, 1 μM, Sigma, St. Louis, MO, USA), an inhibitor of cell division, was added to all cultures from day 1 to obtain glia-free cultures, and half the culture media was exchanged twice a week.

We have previously published that our cultured sympathetic ganglion neurons express markers and properties of sympathetic neurons, including expression of the vesicular monoamine transporter, VMAT (Vega et al., 2010), and TrkA (Lockhart et al., 1997), transcription factors associated with sympathetic neuron development (Moon and Birren, 2008), and that they require NGF for survival (Yang et al., 2002; Enes et al., 2020). We have shown that the neurons release norepinephrine in response to evoked activity (Lockhart et al., 1997), and that they switch from noradrenergic to cholinergic signaling in response to known sympathetic cholinergic differentiation factors (Slonimsky et al., 2003), a property that has been extensively described for sympathetic neurons in culture (Furshpan et al., 1986) and *in vivo* (Schotzinger and Landis, 1988; Kanazawa et al., 2010). Sympathetic neurons are known to be able to form cholinergic synaptic connections to each other in culture (O’Lague et al., 1974) and have been used as a model of cholinergic transmission in the sympathetic ganglia (Gingras et al., 2002). We have shown that our cultured sympathetic neurons form such connections while continuing to release norepinephrine (Slonimsky et al., 2003), providing an opportunity to examine synaptic properties of the SHR neurons in culture.

*Primary cardiomyocyte cultures* were generated from isolated left ventricles, divided into ~3 mm long, cubic pieces, and digested for 1 h at 37°C with Collagenase Type II (350 units/ml, Worthington Biochemical Corporation, Lakewood, NJ, USA) in ADS dissociation solution (in mM: 116 NaCl, 20 HEPES, 1.0 NaH_2_PO_4_, 5.5 Glucose, 4.2 KCl, 0.4 MgSO_4_). During the tissue digestion, the enzyme solution was replaced with fresh solution every 20 min. Cells were then dissociated by trituration with fire polished glass pipettes in three rounds of progressively smaller tip sizes. Each round was done in 2 ml of fresh ADS, and only dissociated cells from the second and third rounds were collected. Collected cells were pre-plated onto uncoated tissue culture dishes and received sequential additions of CaCl_2_ every 4 min, to achieve the following concentrations: 50 nM, 100 nM, 200 nM, 500 nM, and 1.0 μM. After the last addition of CaCl_2_, cells were incubated for 1 h at 37°C. After pre-plating, the less adherent cardiomyocytes were removed from the dishes and plated at a density of 35,000 cells/dish on glass-bottomed dishes (MatTek Corporation, Ashland, MA, USA) coated with collagen (50 μg/ml; BD Biosciences, Bedford, MA, USA) and mouse laminin (5 μg/ml; BD Biosciences, Bedford, MA). For co-cultures with neurons, cardiomyocytes were mixed with dissociated neurons before plating, and plated together at the same density of cells/dish as the unmixed cultures (i.e., 10,000 neurons and 35,000 cardiomyocytes per dish). Cultures of cardiomyocytes alone and co-cultures of cardiomyocytes and neurons were maintained as described for the neuronal cultures.

### Electrophysiology

Whole-cell patch-clamp recordings were obtained on cultured neurons using an Axopatch 200 B amplifier (Molecular Devices, San Jose, CA, USA) as previously described (Enes et al., 2020) and as used to measure sympathetic synaptic properties and neuronal excitability in other studies (Xia et al., 2002; Jia et al., 2008; Luther et al., 2013; Davis et al., 2022). Briefly, dishes were continuously perfused with extracellular solution (in mM: NaCl 150, KCl 3, CaCl_2_ 2, MgCl_2_ 2, HEPES 10, and D-glucose 11; pH 7.4 and adjusted to 320 mOsm with sucrose) using a gravity-based perfusion system. All recordings were made at 33–35°C using an SH-27B in-line solution heater and QE-1 heated culture dish platform (both from Warner Instruments, Inc., Hamden, CT, USA) held on the platform stage of an Olympus IX70 microscope. Patch pipettes pulled on a P-97 horizontal micropipette puller (Sutter Instruments, Novato, CA, USA) using borosilicate glass capillaries (1B100F-4; World Precision Instruments, Inc., Sarasota, FL, USA) had resistances of 3–5 MΩ and were filled with an internal solution containing, in mM: K gluconate 100, KCl 30, MgSO_4_ 1, EGTA 0.5, HEPES 10, K_2_ATP 2, NaGTP 0.3, Tris phosphocreatine 10; pH 7.2 and adjusted to 290 mOsm with sucrose. Data were acquired with pClamp 8 software suite, digitized at 10 kHz by DIGIDATA 1320A (Molecular Devices, San Jose, CA, USA), and low-pass filtered at 2 kHz. Electrophysiological responses were analyzed using built-in functions in MATLAB (The MathWorks, Inc., Natick, MA, USA).

Spontaneous activity was recorded for 5 min at a holding potential of −60 mV; cells were classified as silent if they showed fewer than 10 single events in the 5 min period. Silent cells were excluded from further analysis. The total synaptic charge was defined as the area above the 25 pA threshold signal, i.e., the sum of all the current values above 25 pA. Average synaptic charge corresponds to total synaptic charge per 10 s duration. Values presented in plots are average membrane currents quantified as averaged synaptic charge normalized to 1 ms duration. Due to incomplete voltage clamp, we occasionally found cells with activity that showed escaping action potentials identifiable based on an amplitude >1 nA and duration <7.5 ms. Those spikes were excluded from the quantification by cutting them off from the original trace and replacing them with interpolated values. Series resistance (R_s_) was monitored throughout recordings but not compensated. Cells were accepted for analysis only if they met the following criteria: (a) resting Vm < −45 mV (not corrected for liquid junction potential); (b) R_series_ < 20 MΩ; and (c) R_input_ >100 MΩ and not varying more than 20% of the initial value over the course of the recording.

An evoked activity was recorded in an extracellular solution containing the nicotinic cholinergic antagonist hexamethonium bromide (100 μM; Sigma, St. Louis, MO, USA). A small dc current was injected to maintain membrane potential at −60 mV in between depolarizations. To examine the firing properties, incremental step current pulses of 500 ms duration were injected into the cell typically repeated every 4 s. The average cell response was calculated from three consecutive trials.

### Immunocytochemistry

Cultured cells were fixed with 4% paraformaldehyde (PFA, Sigma-Aldrich; St. Louis, MO, USA) for 10 min, washed with phosphate-buffered saline (PBS), permeabilized in 0.1% Triton-X 100 (Sigma-Aldrich; Burlington, MA, USA) in PBS for 10 min, and blocked 1 h with 3% bovine serum albumin (BSA) (Jackson ImmunoResearch; West Grove, PA, USA) or 10% donkey serum (Fisher Scientific; Waltham, MA, USA). *For neuronal cultures*, synaptic sites were identified by the co-localization of the pre-synaptic Vesicular Acetylcholine Transporter (VAChT) protein and the post-synaptic Shank protein in Microtubule Associated Protein2 (MAP2) stained neurons. We previously demonstrated co-localization of VAChT with Synapsin 1 in our sympathetic cultures (Vega et al., 2010), and Shank has been shown to colocalize with acetylcholine receptors and PSD93 at sympathetic synapses (Parker et al., 2004), validating our use of these markers for identification of pre- and post-synaptic sites. Primary antibodies used were: rabbit (rb) anti-VAChT (Synaptic systems; Goettingen, Germany, 139103, 1:500), mouse (ms) anti-shank (Millipore; St. Louis, MO, USA, MABN24, 1:250) and chicken (ck) anti-MAP2 (Millipore; St. Louis, MO, USA, AB5543, 1:1,000); secondary antibodies used were: goat (gt) anti-rb Alexa 647 (Invitrogen; Waltham, MA, USA, a32733TR, 1:500), gt anti-ms Alexa 488 (Invitrogen; Waltham, MA, USA, a11029, 1:500) and gt anti-ck Alexa 568 (Invitrogen; Waltham, MA, USA, a11041, 1:500). Primary antibodies were incubated at 4°C overnight and secondary antibodies at room temperature for 1 h. Both sets of antibodies were diluted in 1.5% BSA (Jackson ImmunoResearch; West Grove, PA, USA) with 0.1% Triton X-100 (Sigma-Aldrich; Burlington, MA, USA). *For myocyte-containing cultures*, cardiomyocytes were identified by expression of cardiac troponin T (cTnT, Invitrogen; Waltham, MA, USA, MA5-12960 1:200) and neurons (for co-cultures) with ck peripherin primary (EMD Millipore; Burlington, MA, USA, AB9282, 1:1,000). Secondary antibodies were anti-ms AlexaFluor 594 (Jackson ImmunoResearch; West Grove, PA, US, 715-585-151, 1:500 or 1:250) and anti-ck AlexaFluor 488 (Jackson ImmunoResearch; West Grove, PA, US, 703-545-155, 1:500). Nuclei were stained with 4’,6-diamidino-2-phenylindole (DAPI, 2 μg/ml, Invitrogen; Waltham, MA, USA,) 10 min at room temperature in PBS.

### Image analysis

8-bit images were acquired using a ZEISS LSM 880 Confocal microscope at 1,024 × 1,024 pixel resolution. Images were acquired sequentially under identical settings of laser strength, detector gain, and detector offset across all conditions within each culture.

#### Synapse quantification

Images were acquired using a 63× oil objective. Settings were chosen to exclude signal saturation in each channel using Quick Lookup Tables (QLUT) available in the Leica image acquisition software. The maximum intensity projection of each image was then analyzed using Puncta Analyzer, an ImageJ plugin written by Barry Wark and obtained upon request from c.eroglu@cellbio.duke.edu (Ippolito and Eroglu, 2010). The fibers of the neurons extensively arborize across the dish although the smaller fibers are not captured in our images due to oversaturation of the proximal dendrites and soma. Counting puncta in these proximal dendrites and soma allows us to ensure that we are counting co-localized sites associated with a single neuron. We confirmed neuron-specific staining by demonstrating that, upon overexposure to MAP2, Shank and VAChT puncta are confined to the MAP2-positive fibers. The number and size of synaptic puncta on neuronal cell bodies and proximal dendrites (<50 μm from the soma) were quantified using identical threshold values for all cells in both conditions. The number of synaptic puncta was normalized to the MAP2-positive area. The size of synaptic puncta, defined as the total area of co-localized pre- and post-synaptic markers, was calculated using Puncta Analyzer.

#### Cardiomyocyte area

Images were acquired using a 10× objective. Cardiomyocyte area was determined using an in-house MATLAB code which identifies myocytes by expression of cTnT signal and selects for size measurement only cells that have at least one but no more than two nuclei, as determined by DAPI staining. Measurements were converted from pixels to micrometers *post hoc* in Microsoft Excel, based on units from image metadata.

### Statistics and figures

Results are presented as mean ± s.e.m. Prism software (GraphPad; San Diego, CA, USA) and SigmaStat (Systat Software Inc; San Jose, CA, USA) were used to plot data. Figures were assembled in Adobe Illustrator (Adobe, Inc; San Jose, CA, USA). Statistical analysis was done using Prism software (GraphPad; San Diego, CA, USA). A nested *t*-test was used for experiments involving comparison of two data sets; for multiple comparisons, the appropriate analysis of variance (ANOVA) test (one-way ANOVA with multiple comparisons, 2-way ANOVA with repeated measures, or nested 1-way ANOVA with multiple comparisons) was used, followed by *post hoc* Tukey’s comparisons. For electrophysiological data with non-normal distributions, the non-parametric Kruskal-Wallis test with Dunn’s multiple comparison test was used.

## Results

### Increased synapse formation in cultured SHR sympathetic neurons

**Figure 1:**
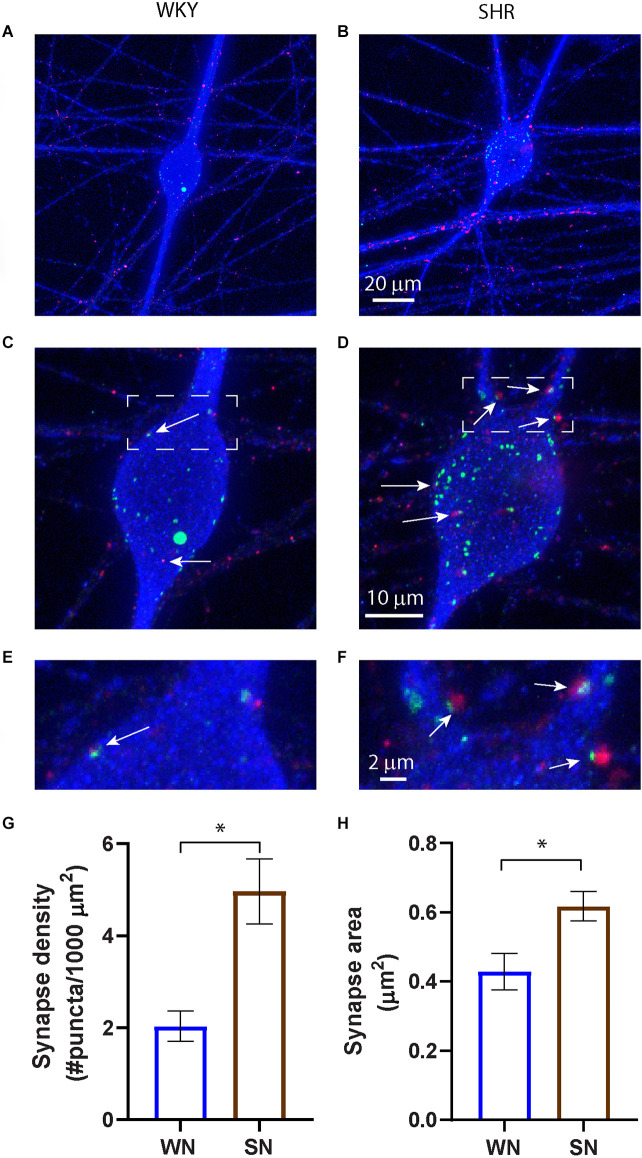
Increased cholinergic synaptic sites in cultured SHR sympathetic neurons. **(A–F)** Images of WKY **(A,C,E)** and SHR **(B,D,F)** neurons at 14 DIV stained for the presynaptic marker VAChT (red), the postsynaptic marker Shank (green), and MAP2 (blue) to stain neurons and dendrites. Overexposure of MAP2 in images **(A)** and **(B)** highlight the localization of synaptic markers to neuronal fibers. Boxed images in **(C)** and **(D)** are enlarged in **(E)** and **(F)** to show co-localization of pre- and postsynaptic markers on the soma and proximal dendrites (white arrows). **(G)** Quantification of co-localized markers on the neuronal soma and proximal dendrites show an increased density of co-localized sites and, **(H)** an increase in puncta size in the SHR compared to WKY neurons (WN, *n* = 113 neurons from *N* = 7 primary culture preparations; SN, *n* = 110 neurons from *N* = 7 primary culture preparations; nested unpaired t-test, *p < 0.001, error bars represent s.e.m.). Scale bars are 10 μm **(A** and **B)**, 20 μm **(C** and **D)**, and 2 μm **(E** and **F)**.

We examined the development of cholinergic synaptic sites on the soma and proximal dendrites of cultured SHR and WKY sympathetic neurons. Neurons were isolated from superior cervical ganglia (SCG) at P2–4, and following depletion of non-neuronal cells (see Section “Methods”), were cultured in the presence of sufficient NGF to support survival for 14 days *in vitro* (DIV14) to allow for the development of synaptic contacts (O’Lague et al., 1974; Luther et al., 2013). Cultures were stained for MAP2 to stain neuronal soma and dendrites and co-stained for the cholinergic presynaptic marker vesicular acetylcholine transferase (VAChT) and for Shank, a postsynaptic marker that is localized to synaptic sites in sympathetic neurons (Parker et al., 2004; Vega et al., 2010; Enes et al., 2020). We confirmed that stained puncta localized to neuronal fibers (Figures 1A,B) and counted the number of Shank and VAChT-positive puncta on the soma and proximal dendrites of individual neurons (Figures 1C–F). The density of putative synaptic sites was determined as the number of co-localized puncta normalized to the MAP2-stained area (Figure 1G). SHR neurons had a higher density of co-localized sites compared to WKY neurons, suggesting a larger number of active cholinergic synapse sites. The size of the synaptic sites was also greater in SHR neurons, as shown by analyzing the total number of pixels in the co-localized sites and converting pixels to area (Figure 1H). These data suggest that in the absence of local non-neuronal signaling and at low NGF concentrations the synaptic properties of SHR sympathetic neurons are enhanced compared to the WKY neurons at an early developmental timepoint.

### Increased synaptic transmission in cultured SHR compared to WKY sympathetic neurons

**Figure 2:**
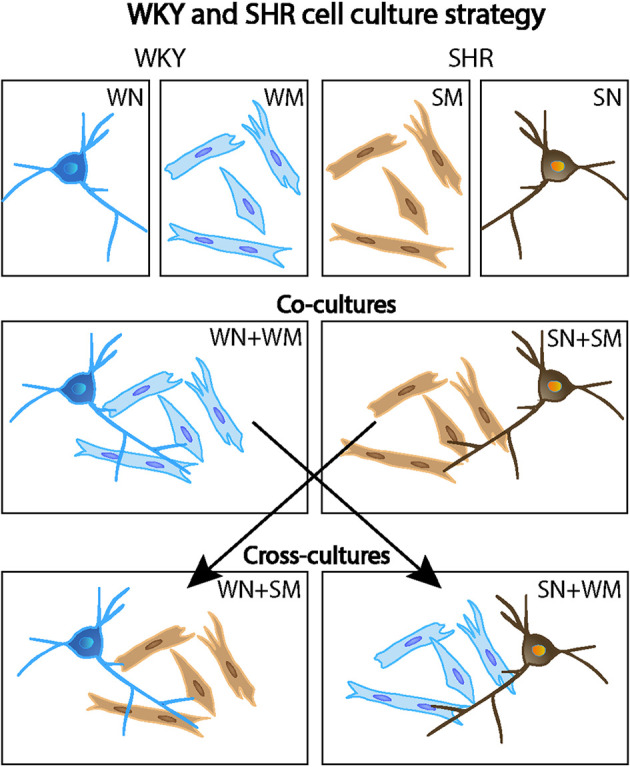
Mixed co-culture experimental design. Primary cultures of WKY and SHR were grown alone (top row: WN, WKY neurons only; WM, WKY cardiomyocytes only; SM, SHR cardiomyocytes only; and SN, SHR neurons only). In some experiments, co-cultures of neurons and cardiomyocytes from the same rat strain were used (middle row: WN + WM, WKY neurons, and WKY cardiomyocytes and; SN + SM, SHR neurons and SHR cardiomyocytes). Cross-cultures conditions mixed neurons and cardiomyocytes from the two different strains (bottom row: WN + SM, WKY neurons, and SHR cardiomyocytes and; SN + WM, SHR neurons, and WKY cardiomyocytes. This cell culture strategy allowed analysis of strain-specific contributions of individual cell types to neuronal activity and cardiomyocyte size.

We examined the spontaneous synaptic activity of SHR and WKY neurons and asked if cardiomyocytes, a target of sympathetic innervation, influenced synaptic transmission of WKY and SHR neurons. Sympathetic neurons were isolated from P2-4 rats and cultured for 18–24 DIV to allow the development of functional cholinergic synaptic transmission. As shown in Figure 2, WKY or SHR neurons were grown alone (WN or SN) or co-cultured with cardiomyocytes from the same strain (WN + WM and SN + SM). We also asked if the effects of cardiomyocytes differed between strains by cross-culturing SHR neurons with WKY cardiomyocytes (SN + WM) and WKY neurons with SHR cardiomyocytes (WN + SM) (Figure 2).

**Figure 3:**
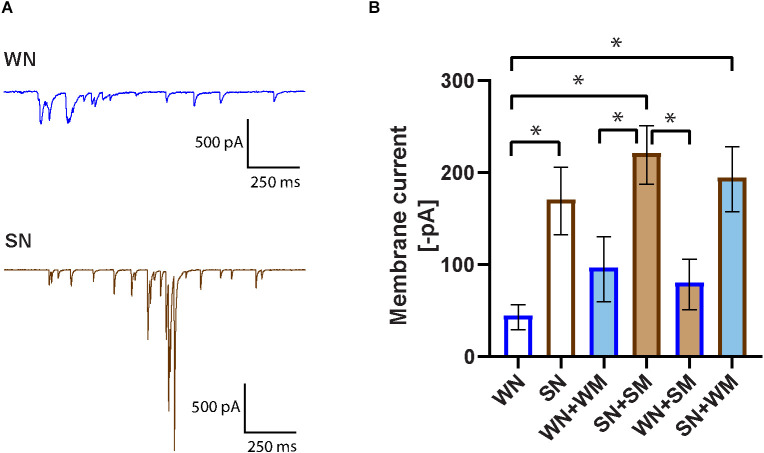
Increased spontaneous activity in SHR neurons. **(A)** Representative voltage-clamp recordings of spontaneous activity of a WKY neuron (WN) and SHR neuron (SN) held in voltage clamp at −60 mV. **(B)** Synaptic activity was quantified as membrane current (see “Methods” Section) for neurons cultured for 18–24 DIV. Bar outline denotes neuron type, fill color denotes cardiomyocyte type. Bars without fill, neurons only; dark blue line, WKY neurons; dark brown line, SHR neurons. Light blue fill, WKY cardiomyocytes; light brown fill, SHR cardiomyocytes, WN, *n* = 30 neurons from *N* = 17 primary culture preparations; SN, *n* = 27, *N* = 14; WN + WM, *n* = 24 *N* = 18; SN + SM, *n* = 30, *N* = 15; WN + SM, *n* = 26, *N* = 15; SN + WM, *n* = 28, *N* = 9; non-parametric Kruskal-Wallis test with Dunn’s multiple comparison test, *p < 0.05, error bars represent s.e.m.

Whole-cell voltage-clamp recordings were obtained on the neurons and total synaptic charge, measured as the average of current values above 25 pA over a 10 s period, was quantified. A comparison of SHR (SN) and WKY (WN) neurons found a higher synaptic charge in the SHR neurons (Figures 3A,B). We next measured the synaptic charge of SHR neurons co-cultured with cardiomyocytes derived from the same animals (SN + SM) and WKY neurons cultured with WKY cardiomyocytes (WN + WM). Similar to the result for neurons cultured alone, co-cultured SHR neurons had a greater synaptic charge than co-cultured WKY neurons (SN + SM vs. WN + WM). There was no significant difference in synaptic charge between co-cultures and neurons grown alone for either strain (SN vs. SN + SM and WN vs. WN + WM), although each type of neuron showed a trend toward increased activity when cultured with the strain-matched cardiomyocytes (Figure 3B).

We tested the idea that compensatory changes between SHR neurons and SHR cardiomyocytes could influence the level of synaptic activity and that SHR neurons might have a different response to WKY cardiomyocytes. We cultured SHR neurons with WKY cardiomyocytes (SN + WM) and WKY neurons with SHR cardiomyocytes (WN + SM). We found that WKY cardiomyocytes did reduce the heightened synaptic activity of SHR neurons in SN + WM cultures and that SHR cardiomyocytes did not increase synaptic charge in WN + SM cultures (Figure 3B). These data suggest an early increase in synaptic activity in SHR neurons that, in our simplified culture system is unmodified by interactions with cardiomyocytes. Together these data support a model in which early cell-autonomous mechanisms contribute to high levels of synaptic transmission in young SHR neurons.

### Firing properties of cultured SHR neurons

**Figure 4:**
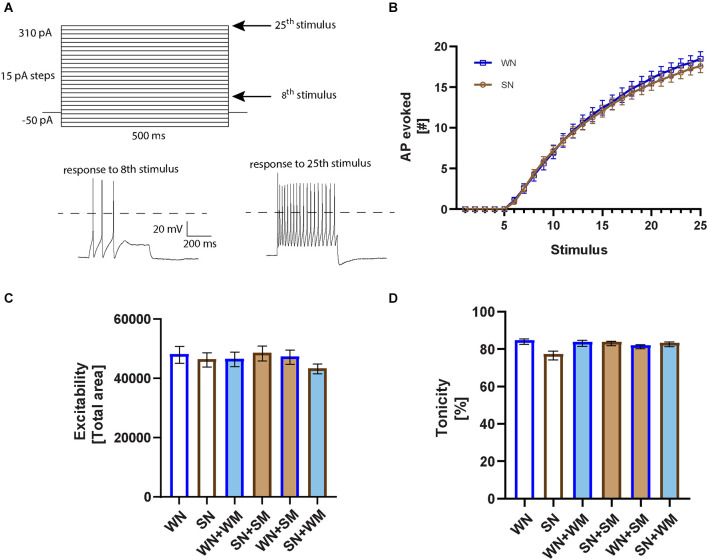
Intrinsic neuronal excitability is not different in WKY and SHR neurons. **(A)** Square pulse stimuli pattern applied in current-clamp to evoke action potential firing. Representative recordings corresponding to the right arrows marking the 8th (55 pA) and 25th stimulus (310 pA) are plotted below. A dotted line is plotted at 0 mV. **(B)** Excitability curves for WKY neurons (WN) and SHR neurons (SN) cultured 2–9 DIV showing the number of evoked action potentials (APs) in response to each of the 25 stimuli; *n* = 26, WN; 37, SN, 2-way ANOVA with repeated measures indicates no significant difference between WKY and SHR neurons. **(C)** The total area under the excitability curves was calculated and averaged for each co-cultured condition (WN, *n* = 26 neurons from *N* = 10 primary culture preparations; SN, *n* = 37, *N* = 15; WN + WM, *n* = 28, *N* = 11; SN + SM, *n* = 30, *N* = 12; WN + SM, *n* = 28, *N* = 9; SN + WM, *n* = 34, *N* = 11; one-way ANOVA, error bars represent s.e.m.). **(D)** Tonic properties of WKY and SHR neurons. Comparison of the number of evoked APs in the first vs. the second half of the 25th stimulus for the neurons recorded from in **(C)**, non-parametric Kruskal-Wallis test with Dunn’s multiple comparison test, error bars represent s.e.m.

We measured the firing rates of SHR and WKY neurons to determine if differences in spontaneous activity were associated with altered intrinsic excitability. We obtained whole-cell current-clamp recordings from cultured neurons grown for 2–9 DIV and measured the number of spikes evoked by depolarizing current steps ranging from −50 to 310 pA with 15 pA increments (Figure 4A). Analysis of the current-firing relationship showed no difference between WKY and SHR neurons (Figure 4B). Neuronal firing was not influenced by co-culture with cardiac myocytes, whether neurons were co-cultured with cardiomyocytes derived from the same strain or cross-cultured with either SHR or WKY cardiomyocytes (Figure 4C).

Sympathetic neurons can fire in either a tonic or a phasic pattern (Jia et al., 2008; Springer et al., 2015). We analyzed the degree of tonic and phasic firing in neurons isolated from P2-4 SHR and WKY rats and cultured 2–9 days to determine if altered patterns of firing were observed as early as the postnatal period. We calculated a tonicity index, defined as 100 × (number of spikes in the second half of the stimulus/number of spikes in the first half of the stimulus), where 100% describes a cell with a fully tonic firing pattern in which spikes are evenly divided between the first and second half of the stimulus, and 0% describes a phasic cell with all spikes only in the first half of the stimulus (Luther and Birren, 2009). There was a small non-significant shift towards phasic firing in the SHR neurons grown in the absence of cardiomyocytes (Figure 4D) that was not seen when the neurons were co-cultured with cardiomyocytes from either the same strain or in mixed co-cultures of SHR neurons with WKY cardiomyocytes. The spiking patterns of WKY neurons cultured with either WKY or SHR cardiomyocytes did not differ from that of WKY neurons grown alone. The finding of similar excitability and tonic firing in both postnatal SHR and WKY neurons is in contrast to studies showing phasic firing in WKY neurons and tonic firing and increased excitability in SHR neurons in cultures from juvenile animals grown in the presence of non-neuronal cells and at high NGF concentrations, suggesting a potential defect in cellular signaling involved in normal developmental down-regulation of firing rates.

**Figure 5:**
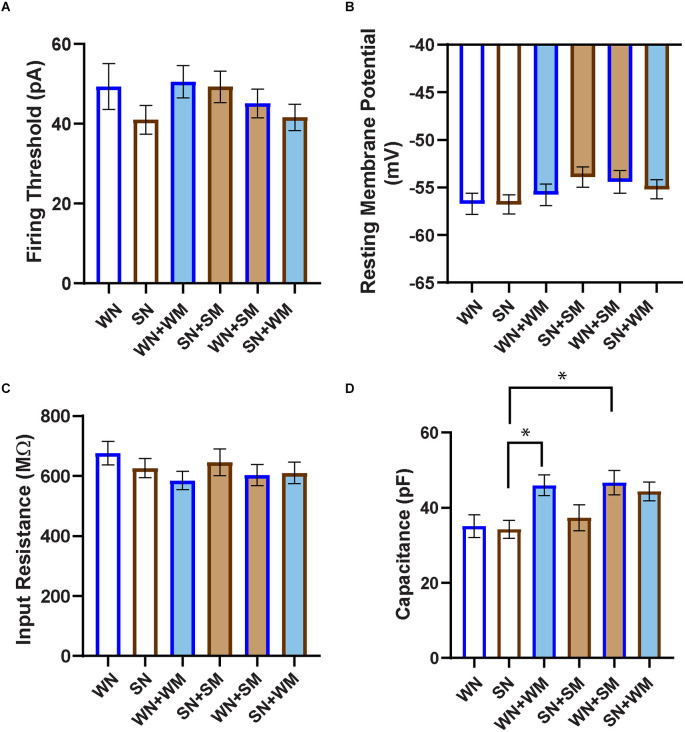
Passive properties of WKY and SHR neurons co-cultured with and without cardiomyocytes. **(A)** The firing threshold was determined in current-clamp mode, and was defined as the smallest current step pulse that evoked at least one action potential for the neuron recordings shown in Figure 4. **(B)** Resting membrane potential was determined immediately after establishing whole-cell recording. **(C)** Input resistance was calculated from the responses obtained at the stable end of applied stimuli, without the presence of nearby APs. No significant difference in firing threshold, resting membrane potential, or input resistance was observed between any co-cultured groups. **(D)** Capacitance was taken as the readout value from the pClamp amplifier after capacitance compensation in the established whole-cell mode. SHR neurons (SN) showed smaller capacitance than WKY N co-cultures with cardiomyocytes from either strain (WN + WM, WN + SM). WN, *n* = 30 neurons from *N* = 17 primary culture preparations; SN, *n* = 27, *N* = 14; WN + WM, *n* = 24, *N* = 18; SN + SM, *n* = 30, *N* = 15; WN + SM, *n* = 26, *N* = 15; SN + WM, *n* = 28, *N* = 9; non-parametric Kruskal-Wallis test with Dunn’s multiple comparison test, *p < 0.05, error bars represent s.e.m.

We further characterized the passive membrane properties of the SHR and WKY neurons. We measured the threshold for action potential firing, input resistance, and resting membrane potential in neurons isolated from P2-4 WKY and SHR animals and cultured for 2–9 DIV. We found no difference in these properties (Figures 5A–C) between WKY and SHR neurons and saw no effect of co-culture with cardiomyocytes from the same or from a different strain. We also measured capacitance under all conditions. There was no difference in capacitance between SHR and WKY neurons cultured alone (Figure 5D). There was a trend towards increased capacitance when either WKY or SHR neurons were cultured with cardiomyocytes from either strain. The capacitance of SHR neurons grown alone was significantly different than WKY neurons cultured with either WKY or SHR cardiomyocytes, but not from SHR neurons grown with any type of cardiomyocyte. These data suggest that WKY neurons may have a greater sensitivity to cardiomyocyte-derived signals affecting cell size compared to SHR neurons.

We tested whether differences in intrinsic excitability developed after a longer culture period, corresponding to the time at which we observed changes in synaptic properties. We found an overall decrease in the firing rates of both SHR and WKY neurons at 14–28 DIV compared to the early time points. The total area under the excitability curve was: WN, 48,241 ± 2,850 at DIV 2–9 (see Figure 4C, *n* = 26); WN, 25,974 ± 1,870 at DIV 14–28 (*n* = 26 neurons); SN, 46,481 ± 2,386 at DIV 2–9 (Figure 4C, *n* = 37); SN, 25,928 ± 1,637 at DIV 14–28 (*n* = 21). This decrease over time in culture is consistent with developmental changes in potassium channels that have been observed in postnatal sympathetic cultures (McFarlane and Cooper, 1993). We did not observe differences in the firing rates in neurons between the two strains at any time in culture, suggesting that synaptic changes that we observed in postnatal SHR neurons are independent of the intrinsic excitability of the neurons.

### Developmental differences in cardiomyocyte hypertrophy

**Figure 6:**
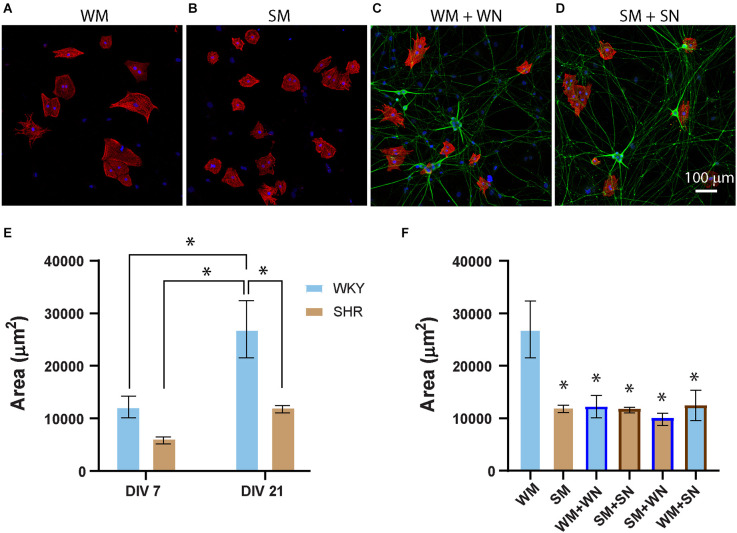
SHR cardiomyocytes are smaller than WKY, and less responsive to neuronal signaling. **(A–D)** Representative images of WKY **(A,C)** and SHR **(B,D)** cardiomyocytes at DIV 7, grown alone **(A,B)** or with neurons from the same strains **(C,D)**, and stained for the myocyte marker cTnT (red) and the neuronal marker peripherin (green). **(E)** Quantification of cardiomyocyte area for cardiomyocytes grown alone (WM and SM) at DIV7 and DIV21 showing a greater size of WM, compared to SM at DIV 21 and an increase in WKY size between DIV 7 and DIV 21. **(F)** Comparison cardiomyocytes grown alone (as shown in **E**) with same-strain (WM + WN; SM + SN) and cross-strain (WM + SN; SM + WN) neuronal co-cultures at DIV 21. DIV 21 WM grown alone are significantly larger than cardiomyocytes in any co-culture condition. There are no differences in size between SM grown alone and the same cardiomyocytes grown in any co-culture (DIV 7: WM, *n* = 186 cells from *N* = 2 primary culture preparations; SM, *n* = 352 cells from *N* = 3 primary culture preparation. DIV21: WM, *n* = 101, *N* = 3; SM, *n* = 204, *N* = 4; WN + WM, *n* = 51, *N* = 4; SN + SM, *n* = 200, *N* = 4; WM + SN, *n* = 129, *N* = 3; SM + WM, *n* = 52, *N* = 3. Nested 1-way across all conditions with Tukey’s *post-hoc* analysis, *p < 0.001, error bars represent s.e.m.), scale bar = 100 μm.

Signaling between cardiomyocytes and sympathetic neurons is bidirectional, with sympathetic neurons limiting the growth properties of neonatal cardiomyocytes while driving hypertrophy at later developmental times (Kreipke and Birren, 2015). We, therefore, asked if postnatal SHR cardiomyocytes displayed strain-specific differences in hypertrophy or altered growth in response to neuronal contact. Cardiomyocytes from P2-4 SHR and WKY rats were cultured for 7 and 21 DIV. Cultures were stained for cTnT and DAPI (Figures 6A–D) and cell area was measured for mono- and binucleated cells as previously described (Kreipke and Birren, 2015). Cultured SHR cardiomyocytes showed a trend towards a smaller cell area compared to WKY cardiomyocytes at 7 DIV that reached significance by 21 DIV (Figure 6E). WKY cardiomyocytes increased in size between 7 and 21 DIV, with a smaller increase for SHR cardiomyocytes that did not reach significance. The size difference between postnatal SHR and WKY cardiomyocytes grown in the absence of innervating neurons suggests that there are intrinsic differences in the structural properties of these sympathetic targets several weeks before the onset of hypertension in the SHR.

Regulation of postnatal cardiomyocyte size by co-cultured sympathetic neurons is mediated by β-adrenergic signaling (Kreipke and Birren, 2015). We asked whether this co-culture influenced the size of WKY or SHR cardiomyocytes by comparing cardiomyocytes grown alone with cardiomyocytes cultured with neurons from the same or the different strain (Figure 6F; WM + WN; SM + SN; SM + WN; WM + SN). Culture with neurons from either strain significantly limited the growth of WKY cardiomyocytes at 21 DIV (Figure 6F). In contrast, neurons, irrespective of strain, had no effect on the size of SHR cardiomyocytes at either time point (Figure 6F). Together, these results demonstrate that sympathetic neurons act to limit the growth of postnatal cardiomyocytes derived from neonatal WKY rats, but that SHR neurons lack responsiveness to this neuronal signaling.

## Discussion

Sympathetic drive to the periphery depends upon the normal development and function of ganglionic sympathetic neurons. Development of this system takes place through a set of coordinated processes that include the patterning of target organ innervation (Habecker et al., 2008), the establishment of neurotransmitter identity and release properties (Furshpan et al., 1986; Lockhart et al., 1997), and the excitability and synaptic properties of sympathetic neurons (Luther and Birren, 2009; Luther et al., 2013; McKinnon et al., 2019). Disruption in these early processes has the potential to contribute to the chronic increases in the sympathetic drive that precede and contribute to the development of hypertension in humans and in animal models (Shanks et al., 2013; Grassi et al., 2015). Here we show structural and functional differences in cellular components of the sympathetic-cardiac circuit of SHR and WKY rats during the early postnatal period, under conditions of minimal extrinsic signaling. We observed changes in cardiomyocyte hypertrophy and in neuronal synaptic properties. We also noted tonic firing of SHR neurons, as observed in other studies (Jubelin and Kannan, 1990; Davis et al., 2020). Surprisingly, similar tonic firing was also observed in the WKY cultures, suggesting that neonatal SHR neurons may have altered responses to extrinsic cues that normally reduce WKY firing rate during normal development and in more complex cultures. In addition, a potentially reduced response to cellular signals is also seen for SHR cardiomyocytes, which do not alter their size in response to neuronal contact. These early differences in cellular properties exist several weeks before the onset of hypertension in the SHR, demonstrating that mechanisms that drive increased sympathetic output in prehypertensive rats are established early in the postnatal period.

Postganglionic sympathetic neurons are activated by central preganglionic inputs in the spinal cord (McLachlan, 2003; Coote and Chauhan, 2016). The altered central activity contributes to increased peripheral sympathetic drive, such as is seen in the SHR (Briant et al., 2014). Ultimately, however, the level of sympathetic drive to peripheral organs is determined by the input-output properties of the ganglionic neurons (Springer et al., 2015; McKinnon et al., 2019), which are modulated by peripheral factors (Luther et al., 2013; Enes et al., 2020). This modulation is likely to be altered in hypertensive SHRs, as is seen in whole ganglion recordings of compound action potentials that show increased output in SHR, compared to WKY rats (Magee and Schofield, 1994). We have shown that postnatal SHR neurons have an increased capacity to form synaptic sites, and that these sites are associated with increased synaptic charge in an *in vitro* model for cholinergic sympathetic synapses (O’Lague et al., 1974; Gingras et al., 2002). Although silent cells were excluded from our analysis, we found that the percentage of silent cells was lower in the SHR neuron cultures (25.6%) than in the WKY neurons (49.9%), consistent with a shift towards higher synaptic activity in the SHR. Further, co-culture with cardiomyocytes from either strain reduced the number of silent neurons even further for the SHR neurons (2.7% with SHR cardiomyocytes, 3.7% with WKY cardiomyocytes), with a smaller effect on the WKY neurons (34.7% with WKY cardiomyocytes, 48.7% with SHR cardiomyocytes). These findings suggest that regulation of synapse formation may underlie altered output in the SHR and show that changes in synaptic capacity take place early in SHR development. Further, synaptic changes in purified young SHR sympathetic neurons are independent of contact with cardiomyocyte targets, whether derived from the same strain or from the normotensive WKY rats. This implies that altered synaptic plasticity is an intrinsic property of SHR neurons from an early development stage.

Although SHRs first show increased blood pressure at 6–8 weeks of age, sympathetic neurons from 4 to 5 week old SHR animals have increased excitability and excessive tonic firing rates compared to neurons from control animals (Davis et al., 2020, 2022). It was therefore surprising that we did not see a difference in firing rate between SHR and WKY neurons in our neonatal cultures. The differences in our findings can be accounted for by tonic firing of the WKY neurons, since SHR neurons showed consistently high firing rates across all studies. A possible explanation for our results is our use of an experimental paradigm that minimizes extrinsic signaling. We previously showed that a high concentration of NGF (50 ng/ml) promotes phasic firing, with tonic firing observed at a lower concentration (5 ng/ml) that was sufficient to support neuronal survival (Lockhart et al., 1997; Luther and Birren, 2009). In addition, co-culture with sympathetic satellite glial cells increases NGF levels in sympathetic cultures and results in a downward trend in neuronal excitability (Enes et al., 2020). This raises the interesting possibility that the high concentrations (50–100 ng/ml) of NGF and the presence of ganglionic non-neuronal cells drive phasic firing of normal neurons, while a loss of responsiveness in SHR neurons results in the maintenance of a highly excitable tonic state. Additional work will be needed to test these possibilities, but the idea that the excitability of cultured sympathetic neurons is normally regulated by interactions with non-neuronal cells is supported by work demonstrating the modulation of multiple potassium channels by ganglionic cells (McFarlane and Cooper, 1993). A requirement for these cellular interactions could also explain why we see little effect of co-culture of cardiomyocytes alone on the excitability of our neurons. We cannot rule out the possibility that differences in excitability also reflect differences in development or the properties of stellate ganglion (Davis et al., 2020, 2022) and the SCG neurons used in this and previous studies (Luther and Birren, 2009; Luther et al., 2013). However, a study showing that neonatal WKY SCG neurons cultured in the presence of high NGF and non-neuronal cells also demonstrate phasic firing properties (Jubelin and Kannan, 1990) supports a model of extrinsic modulation of excitability.

Together, our experiments demonstrate a difference in synaptic properties, but not firing rates between WKY and SHR neurons. This could reflect a developmental delay in excitability changes in the SHR neurons, or differential signaling in the modulation of these processes. In this respect, it is interesting that developing sympathetic neurons are in contact with satellite glia as early as embryonic day 16 (Hall and Landis, 1992; Mapps et al., 2022), and glial interactions drive synaptic changes in neonatal sympathetic neurons, with a lesser effect on excitability (Enes et al., 2020). The timing of glial interactions is in contrast to the sympathetic innervation of the heart and other organs, which is incomplete during late embryonic development (Sripanidkulchai and Wyss, 1987; Glebova and Ginty, 2004), and expands over the postnatal period (Habecker et al., 2008). That means that our neonatal sympathetic neurons have had a low level of target contact at the time they are isolated from our cultures. Recent evidence suggests that direct contact with cardiomyocytes provides NGF that supports the survival and maturation of the neurons (Dokshokova et al., 2022) and neurotrophic signaling has been directly linked to a reduction in firing rate and a transition to phasic firing in postnatal neurons (Luther and Birren, 2009). Together, these findings suggest distinct developmental periods for synaptic and firing rate changes that could account for a delayed transition to phasic firing in the postnatal WKY neurons. Additional experiments will be needed to uncover how glial and neurotrophic signals interact to establish neuronal properties and how disruptions can result in disease states.

The development of the peripheral sympathetic-cardiac circuit involves ongoing interactions between innervating neurons and cardiomyocytes in the heart (Kuruvilla et al., 2004; Habecker et al., 2008; Kreipke and Birren, 2015). Sympathetic neurons drive changes in cardiomyocyte properties (Larsen et al., 2016) and pathological hypertrophy has been described in adult hypertensive SHR hearts (Bell et al., 2004). We did not observe this hypertrophy in cardiomyocytes cultured from SHRs during the postnatal period and, in fact, found that cultured SHR cardiomyocytes had a smaller area than age-matched WKY cardiomyocytes. We previously showed that sympathetic neurons decrease the size of neonatal cardiomyocytes in culture *via* a beta-adrenergic mechanism (Kreipke and Birren, 2015). This is a transient developmental effect during a period when immature cardiomyocytes are withdrawing from the cell cycle, and we have shown that the same signaling pathway leads to cardiomyocyte hypertrophy at later times. Our finding of decreased SHR cardiomyocyte hypertrophy is consistent with this earlier study and suggests altered responsiveness, or the timing of that responsiveness, to neuronal signaling. Previous studies of postnatal SHR cardiomyocytes showed hypertrophy associated with growth with ganglionic explants that were unaffected by blockers of sympathetic transmission (Atkins et al., 1992). This suggests that cardiomyocytes may respond to factors released by non-neuronal ganglionic cells in the cultures and is consistent with our finding of altered responses to extrinsic cues. Recent work using stellate ganglia-derived sympathetic neurons from 4 week pre-hypertensive rats shows that hyper-responsiveness of SHR cardiomyocytes to β-adrenergic signaling is promoted by SHR, but not WKY neurons (Larsen et al., 2016). Together, these studies suggest that β-adrenergic-dependent cardiomyocyte pathology in the SHR is driven by neuronal changes, while some structural differences may reflect altered cardiomyocyte responses to neuronal and other signals.

Sympathetic hyperactivity is a hallmark of many cardiovascular disorders, including hypertension and heart failure. However, the relative contributions of sympathetic and cardiomyocyte pathology—and whether their interactions drive the pathological state—remain to be fully described. We showed that early increases in the synaptic capacity of sympathetic neurons is independent of non-neuronal cells in the sympathetic ganglia and of signals from co-cultured cardiomyocytes. Likewise, early pathological changes in myocyte growth may reflect altered responses to neuronal signals. Thus, a series of developmental changes in the SHR sympathetic system precede, by several weeks, the onset of hypertension.

## Limitations

Our experiments cannot rule out the possibility that SCG and stellate neurons develop distinct properties in the SHR at the postnatal developmental stage examined in this study. Matching strain and ganglia in a developmental manner, and accounting for potential diversity in sympathetic neuron subtypes (Li and Horn, 2006), will be important for determining the timing and cell-type specific properties of synaptic properties and excitability in the SHR system.

## Data Availability Statement

The raw data supporting the conclusions of this article will be made available by the authors, without undue reservation.

## Ethics Statement

The animal study was reviewed and approved by Brandeis University Institutional Animal Care and Use Committee.

## Author Contributions

MH, JH, MB, SS, and SBi contributed to the design of the experiments. Experiments were carried out by MH, JH, and MB. MH, JH, MB, SS, SBa, and SBi were involved in data analysis. MH, JH, MB, and SBi prepared the figures and the manuscript and all authors commented on the manuscript during its preparation. All authors contributed to the article and approved the submitted version.

## Funding

This work was supported by National Institutes of Health (NIH) R21NS116316, the Baruchowitz Family Fellowship for Dysautonomia Research, and a grant from the W.M. Keck Foundation.
